# Should we perform systematic electrophysiological study in Steinert's disease?

**DOI:** 10.1186/1749-8090-3-56

**Published:** 2008-10-18

**Authors:** Abdallah Fayssoil

**Affiliations:** 1 Critical care medicine, Raymond-Poincare Hospital, AP-HP, University of Versailles (SQV), 92380 Garches, France

## Abstract

Myotonic dystrophy type 1 (Steinert's disease) is a multisystem disorder with autosomal dominant inheritance. This disease is associated with the presence of an abnormal expansion of a cytosine thymine-guanine (CTG) trinucleotide repeat on chromosome 19q13.3. Because of rhythmic complications, the place for systematic electrophysiological study (EPS) has to be discussed.

## Manuscript

Myotonic dystrophy type 1 (DM1 = Steinert's disease) is a multisystem disorder with autosomal dominant inheritance. This disease is caused by an unstable expansion mutation of the cytosine thymine-guanine (CTG) trinucleotide repeat situated in the 3' non-coding exon of a gene that encodes a serine-threonine protein kinase (DMPK). The incidence of DM1 is estimated to be 1/8000 births [1]. Patients disclose features muscular disorder including myotonia, progressive muscular weakness and wasting associated to a multi-systemic disorder (cataract, hypogonadism, gastrointestinal disorders, and diabetes). Patients with DM1 have a high incidence of sudden cardiac death (SCD) because of ventricular arrhythmias and/or complete heart block [2,3]. Histopathology studies disclose fibrosis in the conducting system and in the sino-atrial node associated with myocyte hypertrophy and fatty acid infiltration.

Groh WJ et al. reported in a mean follow-up period of 5.7 years, 27 sudden deaths in a study including 406 adults' patients with DM1. There is a consensus to explore symptomatic patients with DM1. Electrophysiological study (EPS) is an invasive procedure for assessing conduction abnormalities and risk of SCD (with ventricular stimulation). This procedure should be considered in case of syncope, family history of sudden death, sinus node dysfunction (sinus pause > 3 seconds), ventricular arrhythmias and electrocardiogram (ECG) abnormalities suggesting intra or infrahissian conduction disturbances [4,5]: left bundle branch block, bifascicular or trifascicular block, second or third degree atrio-ventricular (AV) block. Pacemaker implantation should be considered in symptomatic patients with conduction abnormalities. Implantable cardioverter defibrillator (ICD) has proved its efficacy to prevent SCD due to ventricular arrhythmias. An ICD implantation should be considered if spontaneous sustained VT was noted.

In asymptomatic patients, prediction of cardiac events is not easy. About asymptomatic patients, systematic EPS is performed to measure the HV interval and to implant a pacemaker before the occurrence of a high degree AV block if a first degree infra-hissian AV block has been found even if the use of the HV interval to determine the need for pacing is still controversial. Lazarus et al. proposed a prophylactic implantation of a pacemaker when the HV interval is ≥70 ms [4].

Non invasive tests may be used in asymptomatic patients for evaluating the risk of rhythmic complications. ECG may be useful in predicting SCD. In the study reported by Groh WJ et al. [6], MD1 patients with severe abnormality (ECG with at least one of the following features: rhythm other than sinus, PR interval of 240 msec or more, QRS duration of 120 msec or more, or second-degree or third-degree AV block) were at risk for sudden death [6]. Non-invasive ambulatory ECG is another procedure for assessing asymptomatic patients. According to Sá MI. et al., non-invasive ambulatory ECG in patients with myotonic dystrophy type 1 should be performed on a regular basis, at intervals not greater than six months [7]. In a study including 36 patients with myotonic dystrophy type 1, Sá MI et al. found a high prevalence and changes between baseline and follow-up Holter ECG that justified permanent pacemaker implantation in 11 patients [7]. Heart rate turbulence may be considered as a non-invasive risk predictor of ventricular tachy-arrhythmias in Steinert's disease [8]. Hardin et al. [9] investigated the relationship between autonomic nervous system function, using holter monitoring with heart rate variability (HRV) analysis, and the clinical and genetic factors in patients with myotonic dystrophy type 1. The authors found a decline in HRV with increasing age and the precise genetic abnormality. Signal averaged ECG is another non-invasive procedure for analysing risk stratification in asymptomatic patients. The prolongation of total QRS duration on signal averaged ECG in myotonic dystrophy is primarily related to delayed activation of the His and Purkinje tissue and not to the development of true late potentials. Some non-invasive test can predict the occurrence of AV block with the same efficacy than HV measurement to suppress systematic electrophysiological study. According to Babuty D et al. [10], association of QRS duration ≥100 ms with low amplitudes signals (< 40 μV) ≥ 36 ms may identify patients with an abnormal HV interval with good sensitivity (80%) and specificity (83. 3%). Genetic analysis is also a non-invasive assessment of cardiac risk. The severity of cardiac myopathy in DM 1 seems to be correlated with age and CTG repeat length [11]. In the study reported by Babuty D. et al., the size of the DNA mutation was longer in the abnormal HV interval group than in the normal HV interval group (3.5(1.8) *vs *2.2 (1.0) kb, p < 0.02) [10]. For analysing the risk of SCD in asymptomatic patients, there is not a consensus for performing systematic ventricular stimulation. Moreover, even in symptomatic patients it can be difficult to base the decision of ICD implantation on a positive EPS, given the possibility of a false positive result. HV prolongation could be also an indirect marker of risk of ventricular tachycardia.
